# Health systems in India: analysing barriers to inclusive health leadership through a gender lens

**DOI:** 10.1136/bmj-2023-078351

**Published:** 2024-07-17

**Authors:** Jasmine Gideon, Sumegha Asthana, Ramila Bisht

**Affiliations:** 1 Birkbeck, University of London, London, UK; 2 Global Health 5050, Cambridge, UK; 3 Centre for Global Health Science and Security, Georgetown University, Washington DC, USA; 4 Centre for Social Medicine and Community Health, Jawaharlal Nehru University, New Delhi, India

## Abstract

Using India as a case study, **Jasmine Gideon and colleagues** argue that considering how gender perspectives operate within health systems and society can help achieve more inclusive health leadership

In 2019 a World Health Organization (WHO) report laid out vast gender inequalities affecting leadership roles across health systems. Women constitute 70% of the global health workforce but hold only 25% of senior roles.^1^ These global trends are reflected in India, where only 24% of women participate in the formal labour force,^2^ 14.2% of medical doctors are female,^3^ and 28% of leadership roles across national health organisations are held by women.^4^ In India, male medical doctors dominate health leadership despite a scarcity of evidence demonstrating their suitability for guiding healthcare organisations.^5^ How can such imbalances be transformed? This is a central question of the BMJ collection on gender equality in the health workforce ( www.bmj.com/collections/gender-equality-health-workforce). 6 7 8 9

We need feminist forms of leadership that challenge the deep structures of inequalities within the health sector and makes it more socially just.^7^ ^8^ ^10^ Feminist leadership is vital for achieving workplace focused social justice for all people working in health sectors—a concept that encompasses addressing and redressing inequalities, including those of power, privilege, and pay.^8^

Gender imbalance in healthcare leadership is often seen as a linear career progression issue for women, who face a leaky or fractured pipeline. A central contention of the pipeline theory^11^ ^12^ is that women remain concentrated in low status positions because they lack sufficient qualifications to progress; with access to improved qualifications and education, or as greater numbers of women enter the workforce and age, they will gain parity with men, moving on to leadership positions.^11^ ^12^ However, this theory overemphasises individuals and obscures the need for active intervention to remove barriers at structural and organisational levels.^11^ ^12^ Promoting women’s movement along the pipeline without addressing inequalities within the organisation or system risks reinforcing the idea that it is women not systems that require “fixing.”^5^ ^8^ Even when healthcare professions become highly feminised, as has occurred in dentistry, it is not sufficient to overcome the patriarchal institutional structure of the sector, and women continue to face significant barriers to progress.^13^

Instead our starting point is that health systems are not gender neutral.^14^ ^15^ A “gender lens” is a way to “understand how gender power relations create inequities in access to resources, the distribution of labour and roles, social norms and values, and decision-making” in health.^15^ A gendered understanding of health systems also considers the role of unpaid care in health systems and health outcomes. This work is an essential input into health systems yet is rarely acknowledged as a constraint on women’s time or access to power.^14^ However women’s gendered role within the household can limit their participation in paid work and reinforces gendered norms around women’s caring role not only within the household but also more widely across the health system and society. Drawing on research from the Indian context we consider how the gendered structure of the health sector limits women’s access to leadership roles and advocate for change. 

## Effect of national norms, regulations, and financial allocations 

Male power at national level permits elites to shape ideology, norms, and stereotypes as well as formal social institutions so that male activities and traits are seen as superior and more valuable than those of women.^16^ Thus focusing on national level in relation to the health sector facilitates understanding of the importance of the wider political and socioeconomic context in shaping the power of (male) doctors.^17^ The long term failure to challenge medical powers is an important factor that enables the continued medical and male dominance of health leadership in India and elsewhere.^18^

Arguably, the Indian medical model has been shaped and reinforced by the historical exclusion of women from medicine.^19^ Medical roles in India were reserved for men while women were pushed into nursing and midwifery, and despite progress many medical specialties remain male dominated.^7^ Very few women enter cardiology or surgery whereas paediatrics is often considered to be more female friendly and compatible with women’s unpaid caring responsibilities.^20^ Women’s dominance in specialties such as obstetrics and gynaecology mirrors internalised patriarchal values of Indian women, with their career choices reflecting personal preference for female doctors. Moreover, the government has neglected to improve and recognise the professional practices of traditional care givers, including traditional midwives,^21^ resulting in many rural women being excluded from the health system.

Notions of professionalism are rooted in time and place and change over time, reflecting the wider gender regime.^22^ An important part of this regime is the use of occupational closure strategies, which aim for occupational monopoly over the provision of certain skills in a market for services.^23^ Gendered occupational closure strategies enable male power to be used to stake claims to resources and opportunities in labour market.^23^ Within the medical profession these strategies of occupational closure are deeply gendered, racialised, and often class based.^24^ Moreover, the medical profession continues to be rigidly framed around an “ideal” model of a doctor.^25^ Indeed, depictions of the ideal doctor as an “unencumbered worker”^25^ are helpful in understanding the Indian context, where rigid gendered norms around roles and responsibilities persist, constraining women’s progression.^3^ This also highlights one of the limitations of the pipeline argument since it is underpinned by assumptions based on a typical masculine career pattern.^11^

The potential for (male) medical dominance within the health sector is also affected by prevailing economic arrangements. Important factors include the mix of public and private providers alongside the type of payment mechanisms for medical salaries and services provided.^26^ Despite structural reforms to health systems, including increased monitoring of cost effectiveness in health and the expansion of non-state providers, medical dominance within the sector remains strong in many countries. Demand for doctors’ skills remains high and outstrips supply, yet the division of tasks between healthcare workers has not changed, so doctors retain ultimate responsibility for much of medical work in the system.^17^ Within India a series of neoliberal reforms have reduced the role of the state and introduced a range of private organisations into healthcare. These have brought opportunities and challenges for tackling both medical dominance and gendered inequalities in health system leadership.

Although research has highlighted the gendered impacts of different health financing mechanisms for healthcare users,^27^ less attention has been given to their effect on those working within the system. Nevertheless, studies of the effects of cuts in public expenditure on the health workforce in India have shown, in line with wider trends, that they often have the biggest effect on areas of work that are female intensive, cutting salaries and moving workers onto short term contracts. This is common practice in the private sector in India, where hiring underqualified female staff to save costs reinforces gender based hierarchies.^28^

One consequence of a more casualised form of employment is the reduction in employment benefits, including maternity leave, which prevents women from remaining in the workforce. Despite gender equality measures in India, including the Maternity Benefit Act, which requires all employers to protect the maternity rights of employees, the government has failed to regulate the private sector to implement these commitments and places the responsibility of providing maternity benefits on the employer.^29^ Hence, women are more likely than men to be in underpaid or inferior forms of work in the health sector, and this affects equitable inclusion of women in leadership across the sector (box 1).

Box 1 **How national policies produce gendered outcomes**

- In recent years, government health expenditures in India has been 1.2%-1.6% of gross domestic product (GDP).^30^ This low expenditure affects both men and women and limits women’s participation in the public sector workforce or pushes them to work for low wages or on a voluntary basis
- The government does not incentivise or regulate private sector gender equality or enforce the Maternity Benefit Act,^29^ leading to gender and employment inequality
- The government's inadequate investment in public health training institutions 31 has led to the private sector filling the void for education, creating further gaps in training opportunities for women to enter the public health workforce
- The government has failed to recognise and support traditional caregivers in the health system, such as traditional midwives,^21^ leaving many women in rural communities without access to proper healthcare
- The government's promotion of low paid female community health workers to fulfil health education and care roles in the community has reinforced gender hierarchies in health systems 32
- The government is taking steps to empower women in the workforce, including the formation of the Women 20 group,^33^ a 30% increase in the gender budget,^34^ and the introduction of regulatory frameworks to reduce gender disparities in leadership 35

## Organisational policies reinforce gender norms

Gender ideology refers to people’s ideal concept of how to live in the world and reflects a set of hegemonic cultural beliefs about gender. As such, it is normative, justifying the existing social order and the differential roles and rights for women and men.^16^ Consequently, gendered social norms shape institutions and organisations in ways that prioritise men’s employment, promotion, and higher pay. In contrast, unpaid care work is assumed to be primarily women’s responsibility and used as a justification to consider women’s role as secondary and dependent within households and society. Evidence demonstrates that this gender bias within institutions is an important constraint to women’s leadership in the Indian context,^7^ ^36^ while the intersection of ageism and sexism is also particularly prominent in India, limiting women’s access to leadership roles.^37^

Organisational level constraints are considered in more detail elsewhere in this BMJ collection on gender equality in the health workforce,^7^ but here we provide a snapshot of the Indian context where the healthcare model is explicitly underpinned by women’s unpaid and underpaid work. The National Rural Health Mission (subsequently the National Health Mission), established in 2005 with a primary focus on maternal health, created a large (around one million), all female cadre of unpaid community health workers—accredited social health activists (ASHAs). The role was established to improve child health through women speaking to other women in a culturally appropriate manner rather than challenging the patriarchal norms that frame women as primarily responsible for childcare.^38^ Similarly, other frontline healthcare roles such as auxiliary nurse midwives evolved out of gendered norms of caring. The auxiliary midwife programme is rooted in the late 19th century missionary women’s hospitals where midwifery training was started, subsequently providing services in military hospitals. Following Indian independence from colonial rule, these workers were absorbed into rural health services as nurse midwives.^39^ Yet the undervaluing of care work created a lack of professional acceptance of nurse midwives within the system.^40^ Furthermore, gendered power relations that shape the healthcare system, which remains extremely hierarchical, means that nurse midwives are at the bottom of the hierarchy, restricting their access to any form of power.^40^

Although India has introduced policies to improve workplaces for women, they have rarely been implemented effectively. This has been the case with legal frameworks such as India’s Prevention of Sexual Harassment (POSH) Act, introduced in 2013. Despite the regulation, women continue to face workplace sexual harassment in the health sector and there are many barriers to them lodging official complaints, such as fear of being dismissed, losing their reputation, or facing hostility or social stigma.^41^ Box 2 gives some further examples of organisational level constraints to leadership in Indian context.

Box 2 **Some organisational constraints to female leadership**

- Women in India are more often involved in programme specific roles rather than strategic, organisation building roles and decision making,^39^ ^42^ resulting in lack of female role models^43^
- The intersection of ageism and sexism block women’s access to leadership roles^37^
- Men predominate policy conversations, such as the Indian panels at the midwifery congress^18^
- Women in the health sector still lack flexible working hours, childcare facilities at work, and support after maternity leave^35^
- Contractual arrangements and failure to recognise unpaid healthcare workers means female workers are not protected by legislation against sexual harassment^38^

## Family, society, and cultural context 

In India (as elsewhere) women are often assigned, or assumed to be, the primary carers within households and assume responsibility for the unpaid care work needed to maintain a household.^3^ ^44^ Women in India spend 335 minutes a day on unpaid care work while men spend 40 minutes a day^44^ This gendered division of labour influences women’s access to and control over resources, shaping their decision making power. Men’s superior control over resources gives them greater bargaining power to control women’s work and reproductive functions.^16^ Consequently, women’s unpaid care work is often cited as an explanation for why Indian women fail to take on leadership roles^36^ ^37^ or succeed in the competitive world of medicine.^3^ ^37^ ^45^

Gendered social norms push Indian women to prioritise marriage and family over careers. Many therefore leave medicine or take on roles allowing them to combine family responsibilities with work.^3^ ^46^ In a survey of 1607 female dental teachers employed in the 205 dental colleges in India, 63.5% believed their family commitments were barriers to career progression and 64.7% reported that marriage is happier if a man’s career trajectory is superior to his wife’s.^46^ Similarly, female oncologists report difficulties in networking after office hours because of family commitments, constraining their career progression.^47^

## Next steps

Understanding health systems as gendered systems offers critical insights into health leadership inequities. Although Indian women have achieved near parity in enrolment in medical education,^48^ the gendering of health systems in India persists. Despite growing corporatisation and potential new entry points for change, Indian health systems continue to reflect wider and rigid binaries of masculine and feminine, resulting in occupational segregation, with curing roles occupied dominantly by men and caring roles by women. This contributes to a gendered wage gap that favours men, and adverse working conditions and fewer opportunities for women to take leadership positions.

While no single model provides answers to the challenges of gender inequities in Indian health systems, social reforms and government policies have the potential to drive women’s empowerment and gender equality. Effective private sector regulation, reducing historically high gendered literacy gaps,^49^ and dedication to progressive public policies and societal norms, as has occurred in some Indian states, offer inspirations for creating a more gender just health system.^50^ ^51^ Strengthening women’s resilience in negotiating with patriarchy while garnering support from diverse communities can help promote gender equality.^52^ ^53^

We must recognise, value, and support gender inclusivity in healthcare leadership because it not only improves healthcare services but makes them inclusive and equitable places to work. Gender equality in leadership has begun to receive more attention from the Indian government, and Indian chapters of global organisations such as Women in Global Health and WomenLift Health have implemented important strategies for change, especially at the interpersonal level. However, more targeted and robust organisational and national interventions are needed, including effective government regulations to ensure the implementation of affirmative policies. To truly advance gender inclusive health leadership, it is crucial to confront and dismantle the entrenched social norms and expectations, biases, and organisational and structural power imbalances that perpetuate inequality.

**Key messages**

- A gender lens sheds light on the structural, organisational, and interpersonal barriers to gender inclusive health leadership in India, which has lessons for other settings
- Gender inclusivity in healthcare leadership not only improves healthcare services but creates more inclusive workplaces and ensures women’s equality
- The entrenched social norms and expectations, biases, and organisational and structural power imbalances that perpetuate inequality must be dismantled

## Notes

Contributors and sources: JG is a social scientist with over 20 years of experience working on gender analysis of health sector reform and challenges. RB is a social scientist working for over 30 years in public health with special interests in gender, health reforms, and health policy and systems analysis. SA is a health policy and systems researcher with interest in health governance and power asymmetries in health systems. All authors contributed to the conceptualisation of the article. JG and SA wrote the first draft. SA generated the tables. RB acquired data from existing literature. All authors critically revised the manuscript for important intellectual content and contributed equally to further refining, editing, and proofing, and have approved the final version of the article. JG is the guarantor.

Provenance and peer review: Commissioned; externally peer reviewed.

This article is part of the BMJ collection on gender equality in the health workforce (www.bmj.com/collections/gender-equality-health-workforce), developed in partnership with Global Health 50/50, Africa Population and Health Research Centre, and International Center for Research on Women, and funded by the Bill and Melinda Gates Foundation. *The BMJ* commissioned, peer reviewed, edited, and made the decision to publish these articles. The lead editors were Seye Abimbola, Jocalyn Clark, and Emma Veitch for *BMJ Global Health* and *The BMJ*.
